# Beta 2-adrenergic receptor gene association with overweight and asthma in children and adolescents and its relationship with physical fitness

**DOI:** 10.1016/j.rpped.2015.01.012

**Published:** 2015

**Authors:** Neiva Leite, Leilane Lazarotto, Gerusa Eisfeld Milano, Ana Claudia Kapp Titski, Cássio Leandro Mühe Consentino, Fernanda de Mattos, Fabiana Antunes de Andrade, Lupe Furtado-Alle

**Affiliations:** a Universidade Federal do Paraná (UFPR), Curitiba, PR, Brazil

**Keywords:** *ADRB2* gene, Asthma, Overweight, Physical fitness

## Abstract

**Objective:**

To investigate the association of *Arg16Gly* and *Gln27Glu* polymorphisms of β2-adrenergic receptor gene (*ADRB2*) with the occurrence of asthma and overweight and the gene's influence on anthropometric, clinic, biochemical and physical fitness variables in children and adolescents.

**Methods:**

Subjects were evaluated for allelic frequencies of the β2-adrenergic receptor gene, height, weight, body mass index (BMI), BMI *Z*-score, waist circumference (WC), pubertal stage, resting heart rate (HRres), blood pressure (BP), total cholesterol (TC), glucose, insulin, high density lipoprotein (HDL-C), low density lipoprotein (LDL-C), triglyceride (TG), Homeostasis Metabolic Assessment (HOMA2-IR), Quantitative Insulin Sensitivity Check Index (QUICKI) and maximal oxygen uptake (VO_2max_). The participants were divided in four groups: overweight asthmatic (*n*=39), overweight non-asthmatic (*n*=115), normal weight asthmatic (*n*=12), and normal weight non-asthmatic (*n*=40).

**Results:**

Regarding the *Gln27Glu* polymorphism, higher total cholesterol was observed in usual genotype individuals than in genetic variant carriers (*p*=0.04). No evidence was found that the evaluated polymorphisms are influencing the physical fitness. The *Arg16* allele was found more frequently among the normal weight asthmatic group when compared to the normal weight non-asthmatic group (*p*=0.02), and the *Glu27* allele was more frequently found in the overweight asthmatics group when compared to the normal weight non-asthmatic group (*p*=0.03).

**Conclusions:**

The association of *Arg16* allele with the occurrence of asthma and of the *Glu27* allele with overweight asthmatic adolescents evidenced the contribution of the β2-adrenergic receptor gene to the development of obesity and asthma.

## Introduction

Overweight and asthma are chronic diseases that reach alarming prevalence and morbidity.^1^ Excess of weight has been associated with the emergence of respiratory problems such as asthma.^2^ Although the complex interaction between environmental exposure and genetic predisposition to overweight and the development of asthma is not well defined, studies have shown that genetic factors may influence the susceptibility to develop obesity,^3^ and the association between genetic polymorphisms and asthma has been reported.^4^

Beta2-adrenergic receptors (*ADRB2*) are found in several regions of the body, including fat cells, blood vessels, heart and airways.^5^ These receptors play an important role in the genesis of obesity and energy balance regulation. They are responsible for the stimulation of the lipolytic activity in adipose tissue^6^ and for the control of bronchial smooth muscle,^7^ through relaxation and bronchodilation of airway smooth muscle.^8^

The *Arg16Gly* (rs1042713) and *Gln27Glu* (rs1042714) polymorphisms in the *ADRB2* gene seem to be related to the development of overweight, hypertension, metabolic syndrome,^9^^,^ 10 and asthma exacerbations.^11^^,^ 12 They are associated with changes in the sympathetic nervous system activity and may alter lipolysis,^9^ metabolic and cardiovascular regulation,^13^ as well as decrease lung function and bronchodilator response to therapy with β2-agonists.^14^

In children and adolescents, the association between *Arg16Gly* and *Gln27Glu* polymorphisms with obesity or asthma was investigated separately, with controversial results^15^^,^ 16; however, no study has evaluated the frequencies of these polymorphisms in children and adolescents considering obesity and asthma together. Association between *Gln27Glu* polymorphism and physical fitness has been observed only in adults.^17^

The aim of the present study was to investigate the association of the alleles of the *Arg16Gly* and *Gln27Glu* polymorphisms of *ADRB2* gene with the occurrence of asthma and overweight in children and adolescents and to examine whether those polymorphisms influence on anthropometric, clinic, lipid profile and physical fitness variables.

## Method

The sample consisted of 206 children and adolescents of both sexes, 10 to 16 years old from southern Brazil, divided into four groups: overweight asthmatics (*n*=39), overweight non-asthmatics (*n*=115), normal weight asthmatic (*n*=12) and normal weight non-asthmatic (control) (*n*=40). The participants were volunteers from the Pediatric Endocrinology Ambulatory and from public schools of Curitiba, Paraná (Brazil).

The inclusion criteria for overweight participants were: BMI above the 85th percentile of the World Health Organization (WHO)^18^ and, for asthmatic participants, being diagnosed with asthma by medical evaluation and clinical history as recommended by the Brazilian Thoracic Society (Sociedade Brasileira de Pneumologia e Tisiologia -SBPT).^19^ In this study, participants were not classified by the severity of asthma. Exclusion criteria were: presence of diabetes and the use of anorectic drugs or others that may interfere with weight control, insulin levels, blood pressure, glucose or lipid metabolism.

Participants and their guardians signed the Informed Consent Form, according to the protocol approved by the Institutional Review Board of the Federal University of Paraná (CEP/HC2460.067/2011-03).

Height was measured in centimeters (cm) using a stadiometer fixed to the wall (accurate to 0.1 cm), and weight was measured in kilograms (kg), using a digital scale (maximum capacity of 150kg and a resolution of 100g). BMI, expressed in kg/m^2^, was calculated and classified according to the cutoff points for age and sex as proposed by the World Health Organization.^18^ The waist circumference (WC) was measured in centimeters with a flexible and inextensible anthropometric tape (accuracy 0.1cm). Systolic (SBP) and diastolic (DBP) blood pressures were measured after 10 minutes of rest, with the individual seated and with the right arm supported at heart level.

Blood samples were collected in the clinical laboratory in the morning, after 12h of fasting, to perform a complete blood count, measurement of glucose, insulin, total cholesterol (TC), high-density lipoprotein (HDL-C), low-density lipoprotein cholesterol (LDL-C), and triglyceride (TG). The concentrations of TC, HDL-C, TG (mg/dL) and glucose were analyzed by automated enzymatic colorimetric method (CHOD-PAP) (Lab Merck, Darmstadt, Germany; Laboratory Roche, Indianapolis, IN, USA).

Fasting insulin concentration was measured by chemiluminescence immunoassay technique immunometric µU/mL. The HOMA Calculator v2.2 software was used to calculate the insulin resistance, and the Quantitative Insulin sensitivity Check Index (QUICKI), described by Katz et al.,^20^ was used to evaluate insulin sensitivity.

The aerobic fitness analysis was made on the treadmill, and ventilatory responses were measured using a calibrated breath-by-breath monitoring system (Parvo Medics, True Max 2400, Utah, USA), which provided information on oxygen uptake (VO_2_), carbon dioxide production (VCO_2_), pulmonary ventilation (LV), and ratio of respiratory exchange (RER=VCO_2_/VO_2_). These variables were monitored every 15 s. Heart rate was monitored using a heart rate monitor (Polar - model A1, Finland). To ensure that participants had achieved peak oxygen uptake (VO_2max_), at least 2 of the following criteria were observed: (a) exhaustion or inability to maintain the required speed; (b) RER>1.0; (c) maximum heart rate (HR)>190 bpm.

Blood samples (*n*=100) were submitted to leukocyte DNA extraction by a salting-out method^21^ and diluted to the concentration of 20 ng/µL. Variants were genotyped by TaqMan SNP Genotyping Kit (Applied Biosystems) on the device Realplex 2 Mastercycler (Eppendorf). The reaction mix contained 5.0µL of TaqMan Universal PCR Master Mix, 0.5µL of specified TaqMan SNP Genotyping Kit, 2.5µL of ultra-pure water and 2µL of DNA (20ng/µL). Four samples of each genotype were sequenced for method validation.

To verify the normality distribution of data, the Kolmogorov-Smirnov test was used. *T* test and Mann-Whitney test were used for comparisons of means for variables with and without normal distribution, respectively. The allelic frequencies between groups were evaluated by *χ* ^2^tests using Clump software.^22^ Significance was set at a *p*-value <0.05, and the analysis was performed using Statistica for Windows v.10 (StatSoft Inc., Tulsa, EUA)

## Results

The allele frequencies of the *Arg16Gly* and *Gln27Glu*polymorphism of *ADRB2* gene in each of the groups are shown in Table 1. The *Arg16* allele was found with a higher frequency in the normal weight asthmatic group when compared to the normal weight non-asthmatic group (*p*=0.02). For the *Gln27Glu* polymorphism, a significantly higher frequency of the *Glu27* allele was observed in the overweight asthmatic group when compared to the normal weight non-asthmatic group (*p*=0.03), and there was a tendency to significantly higher frequency in the overweight asthmatic group when compared to the overweight non-asthmatic group (*p*=0.05).

**Table 1:** Allelic frequencies of the *Arg16Gly* and *Gln27Glu* polymorphism of *ADRB2* gene among the groups.

Alleles	Overweight/asthmatic *n* (%)	Overweight/non-asthmatic *n* (%)	Normal weight/asthmatic *n* (%)	Normal weight/non-asthmatic *n* (%)
*Arg16Gly*				
Arg 16	26 (37%)	60 (39%)	12 (60%)^a^	22 (31%)
Gly 16	44 (63%)	92 (61%)	8 (40%)	48 (69%)
Total	70 (100%)	152 (100%)	20 (100%)	70 (100%)

*Gln27Glu*				
Gln 27	22 (50%)	98 (66%)	13 (65%)	41 (71%)
Glu 27	22 (50%)^b^	50 (34%)	7 (35%)	17 (29%)
Total	44 (100%)	148 (100%)	20 (100%)	58 (100%)

a Significantly higher frequency compared to normal weight non-asthmatic group (*p*=0.02).

b Significantly higher frequency compared to normal weight non-asthmatic group (*p*=0.03).

The evaluated groups (overweight asthmatic, overweight non-asthmatic, normal weight asthmatic and normal weight non-asthmatic) were pooled to verify the possible effect of the polymorphisms on anthropometric, clinic, lipid profile and physical fitness variables. When separated, according to the *Arg16Gly* site genotypes, into mutation carriers (Arg Gly/Gly Gly) and usual genotype carriers (Arg Arg), the comparisons of the means of anthropometric, cardiorespiratory fitness, blood pressure, biochemical and metabolic profile variables were not significantly different (Table 2).

**Table 2:** Means ± SD of anthropometric, cardiorespiratory fitness, blood pressure, biochemical and metabolic profile variables and comparisons between mutation carriers (Arg Gly/Gly Gly) and usual genotype carriers (Arg Arg) according to the *Arg16Gly* polymorphism site genotypes.

Variables	Arg Arg	Arg Gly/Gly Gly	*t* or *Z*	*p*-value
Age	13.35±1.61	13.00±1.83	0.95	0.34
Weight (kg)	70.76±19.51	68.60±19.48	0.54	0.58
Height (cm)^a^	161.03±9.89	147.43±42.29	1.74	0.08
BMI (kg/m^2^)	26.91±5.89	26.86±6.65	0.03	0.97
BMI *Z*-score^a^	1.96±1.47	2.00±1.51	0.14	0.88
WC (cm)	91.11±17.19	89.94±16.93	0.33	0.73
HRres (bpm)	79.88±9.18	80.15±12.17	−0.10	0.91
VO2max (mL/kg/min)	36.46±7.81	35.16±7.91	0.68	0.49
SBP (mmHg)^a^	108.34±13.08	104.20±12.49	1.87	0.06
DBP (mmHg)^a^	70.68±11.51	68.34±10.27	1.09	0.27
TC (mg/dL)^a^	167.32±36.39	161.79±29.31	0.37	0.70
HDL-C (mg/dL)^a^	47.55±9.73	49.95±12.43	−0.51	0.60
LDL-C (mg/dL)^a^	95.34±31.42	88.69±26.20	0.70	0.48
TG (mg/dL)^a^	115.20±78.23	105.34±61.36	0.11	0.91
Glucose (mg/dL)	88.80±7.10	88.25±7.96	0.31	0.74
Insulin (mU/mL)^a^	12.29±9.20	12.92±9.97	−0.37	0.70
HOMA2-IR^a^	1.57±1.16	1.63±1.21	−0.39	0.69
HOMA2-IR^a^	1.57±1.16	1.63±1.21	−0.39	0.69
QUICKI^a^	0.35±0.04	0.35±0.04	0.39	0.69

BMI, body mass index; WC, waist circumference; HRres; resting heart rate; SBP, systolic blood pressure; DBP, diastolic blood pressure; TC, total cholesterol; HDL-C, high density lipoprotein; LDL-C, low density lipoprotein; TG, triglyceride; HOMA2-IR, Homeostasis Metabolic Assessment; QUICKI, Quantitative Insulin Sensitivity Check Index; VO2max, maximal oxygen uptake.

a Variables without normal distribution.

When separated into mutation carriers (Gln Glu/Glu Glu) and usual genotype carriers (Gln Gln), according to the *Gln27Glu* polymorphism site genotypes, the comparison of the mean and SD of anthropometric, cardiorespiratory fitness blood pressure, biochemical and metabolic profile variables showed no significant differences, except for a significantly higher mean of TC observed in the usual genotype carrier group (Gln Gln) (Table 3).

**Table 3:** Means ± SD of anthropometric, cardiorespiratory fitness, blood pressure, biochemical and metabolic profile variables and comparisons between mutation carriers (Gln Glu/Glu Glu) and usual genotype carriers (Gln Gln) according to the *Gln27Glu* polymorphism site genotypes.

Variable	Gln Gln	Gln Glu/Glu Glu	*t* or *Z*	*p*-value
Age	13.16±2.02	13.29±1.64	−0.42	0.67
Weight (kg)	64.81±19.37	71.63±22.31	−1.91	0.05
Height (cm)^b^	151.74±33.15	148.43±45.50	1.33	0.18
BMI (kg/m^2^)	25.50±6.38	27.10±7.48	−1.34	0.18
BMI *Z*-score^b^	1.69±1.57	1.98±1.64	0.86	0.38
WC (cm)	86.96±17.44	92.38±19.12	−1.76	0.08
HRres (bpm)	77.76±10.38	81.32±10.93	−1.73	0.08
VO2max (mL/kg/min)	35.86±7.22	37.34±6.55	−1.03	0.30
SBP (mmHg)^b^	104.81±13.06	105.02±13.99	0.12	0.90
DBP (mmHg)^b^	70.62±10.59	66.98±10.76	−1.45	0.14
TC (mg/dL)^a^^,^ b	169.23±34.93	158.48±31.51	−1.99	0.04^a^
HDL-C (mg/dL)^b^	50.63±11.99	49.79±12.58	−0.10	0.91
LDL-C (mg/dL)^b^	95.39±33.67	88.20±30.95	−1.40	0.16
TG (mg/dL)^b^	108.67±71.94	102.53±60.71	0.03	0.97
Glucose (mg/dL)	87.72±7.76	87.65±6.76	0.05	0.95
Insulin (mU/mL)^b^	11.85±10.72	12.46±7.94	1.08	0.27
HOMA2-IR^b^	1.50±1.29	1.58±0.98	1.11	0.26
QUICKI^b^	0.36±0.05	0.34±0.04	−1.03	0.30

BMI, body mass index; WC, waist circumference; HRres, resting heart rate; SBP, systolic blood pressure; DBP, diastolic blood pressure; TC, total cholesterol; HDL-C, high density lipoprotein; LDL-C, low density lipoprotein; TG, triglyceride; HOMA2-IR, Homeostasis Metabolic Assessment; QUICKI, Quantitative Insulin Sensitivity Check Index; VO_2max_, maximal oxygen uptake.

a Statistically significant difference.

b Variables without normal distribution.

## Discussion

The prevalence of overweight in the Brazilian population aged between 10 and 19 is 20.5%,^23^ whereas 10.3% of children and 13.8% of adolescents have asthma.^24^ Genetic factors have been demonstrated to contribute to the development of obesity and asthma during childhood, but the roles of specific genes and their interaction are still largely unknown.^3^^,^ 4 ^,^ 15 The A*rg16Gly* and *Gln27Glu*polymorphisms in the *ADRB2* gene have been related to the development of overweight^9^ and asthma exacerbations.^11^^,^ 12

The previously described population frequencies for the *Gly16* allele and for the *Gln27* alleles are 60.4% and 52.7%, respectively.^11^ In obese children and adolescents similar frequencies were found: 59% for *Gly16* allele and 62% for *Gln27* allele.^25^ In a recent study, the allele frequency for *Gly16* in asthmatic children and adolescents was 55%, and 83% for *Gln27*.^26^

The higher frequency of the *Arg16* allele of *Arg16Gly* polymorphism observed in the present study in the asthmatic group, when compared to the control group, suggests that the presence of this allele may be associated with the occurrence of asthma. A tendency to a significantly higher frequency of the *Gly16* allele was observed in the overweight asthmatic group when compared to the normal weight asthmatic group (*p*=0.06), suggesting that the presence of the *Gly16* allele may be related to overweight in asthmatic individuals. Ellsworth et al.^15^ found an association between *Arg16Gly* polymorphism and obesity in male participants: those with Gly Gly genotype showed an increase in BMI during infancy until young adulthood. Different results were found in adolescent girls, showing that carriers of the Gly Gly genotype have a lower probability of obesity than those with Arg Gly or Arg Arg genotypes (*p*=0.006), and girls with Gly Gly genotype have lower BMI compared to those with Arg Arg genotype (*p*=0.049), but higher BMI compared to the Arg Gly (*p*=0.062) genotype.^16^ In another study, female participants who were carriers of the Arg Arg genotype showed higher BMI when compared to those with the Gly Gly genotype.^27^ In the present study, genders were analyzed together due to the small sample size. When classified by usual genotype versus mutation carriers of the *Arg16Gly* polymorphism site, no differences were found for anthropometric variables.

For the *Gln27Glu* polymorphism site, a higher frequency of the *Glu27* allele was observed in the overweight asthmatic group when compared to the control group (*p*=0.03), and there was a tendency toward significance when compared to the non-asthmatic overweight group (*p*=0.05), suggesting that the *Glu27* allele may be involved in the development of asthma in overweight individuals. Large et al.^9^ found the *Glu27*allele associated with obesity, and Ochoa et al.^28^ found no association between *Gln27Glu* polymorphism and obesity in boys, but in female carriers of the *Glu27* allele they found a higher risk of obesity. Research has shown that African American girls who are carriers of the Glu/Glu genotype have higher mean WC than girls without the allele, an association not found among boys.^29^ In the present work, when separated by usual genotype and mutation carriers for the *Gln27Glu* site, no differences were found for anthropometric variables.

There is evidence that adrenoceptor genetic variants have a role in the physiopathology of hypertension, but the results on the relationship between the *ADRB2* polymorphisms are still discordant.^10^ A study from Chou et al.^16^ found an association of the *Arg16Gly* polymorphism with hypertension in obese adolescents, showing that carriers of the *Gly*/*Gly* genotype have a lower probability of hypertension than patients with Arg/Gly or Arg/Arg genotypes (*p*=0.005). No other study so far associated *ADRB2*polymorphisms and blood pressure in children and adolescents. In the present study, no evidence was found that the evaluated polymorphisms are acting on the blood pressure variables.

Adrenergic receptors play an important role in the lipolysis regulation and energy expenditure, so it is possible that polymorphisms in these genes contribute to the emergence of metabolic changes.^30^ A Brazilian study with obese children did not find differences in metabolic and biochemical variables (glucose, TC, LDL-C, HDL-C, TG, leptin, insulin, glucose area and insulin, and HOMA-IR) between haplotypes for *Arg16Gly* and *Gln27Glu* polymorphisms.^13^ In the present work, when separated by usual genotype versus mutation carriers of the *Arg16Gly* polymorphism site, no differences were found for metabolic and biochemical variables. As for the *Gln27Glu*polymorphism site, the usual group showed higher mean TC value when compared to the mutation carrier group.

The association of the *Arg16* allele with the occurrence of asthma and of the *Glu27* allele with the development of asthma in overweight individuals evidenced the contribution to the development of obesity and asthma during childhood and adolescence. The possible association between studied polymorphisms and gender as well as the severity of the disease cannot be evaluated, since the division of the patients into four groups reduced the number of each, and this was the main limitation of the study. Further studies with larger sample sizes may allow the differentiation of groups by gender, as well as the implementation of more robust statistical analysis regarding the roles of the *Arg16*and *Glu27* alleles in childhood overweight/obesity and asthma. The evaluated polymorphisms seem not to influence on the physical fitness in childhood and adolescence.
