# Supplementary material for: Beta 2-adrenergic receptor gene association with overweight and asthma in children and adolescents and its relationship with physical fitness
Source: Rev Paul Pediatr. 2015 Oct-Dec;33(4):381–6. doi: 10.1016/j.rpped.2015.01.012 (PMC4685556; doi:10.1016/j.rpped.2015.01.012)
Supplement: Translation

# Translation: Associação do gene ADRB2 com sobrepeso e asma em crianças e adolescentes e sua relação com a aptidão física

**Authors:** Neiva Leite, Leilane Lazarotto, Gerusa Eisfeld Milano, Ana Claudia Kapp Titski, Cássio Leandro Mühe Consentino, Fernanda de Mattos, Fabiana Antunes de Andrade, Lupe Furtado-Alle

**Affiliations:** a Universidade Federal do Paraná (UFPR), Curitiba, PR, Brasil

**Keywords:** Gene *ADRB2*, Asma, Sobrepeso, Aptidão física

## Abstract

**Objetivo:**

Investigar a associação dos polimorfismos *Arg16Gly* e *Gln27Glu* do gene receptor β2-adrenérgico (*ADRB2*) com a ocorrência de asma e sobrepeso, bem como a influência do gene sobre variáveis antropométricas, clínicas, bioquímicas e de aptidão física em crianças e adolescentes.

**Métodos:**

Os indivíduos foram avaliados quanto à frequência alélica do gene *ADRB2*, estatura, peso, índice de massa corporal, IMC-escore Z, circunferência abdominal, estágio puberal, frequência cardíaca de repouso, pressão arterial, colesterol total, glicose, insulina, lipoproteína de alta densidade, lipoproteína de baixa densidade, triglicerídeos, *Homeostasis Metabolic Assessment*(HOMA2-IR), *Quantitative Insulin Sensitivity Check Index*(QUICKI) e consumo máximo de oxigênio (VO_2max_). Os participantes foram divididos em quatro grupos: sobrepeso asmático (n=39), sobrepeso não asmático (n=115), peso normal asmático (n=12) e peso normal não asmático (n=40).

**Resultados:**

Com relação ao polimorfismo *Gln27Glu*, foi observado maior valor de colesterol total nos indivíduos do genótipo usual do que naqueles que carregam a variante (*p*=0,04). Não foi encontrada evidência de que os polimorfismos avaliados influenciem a aptidão física. O alelo *Arg16* foi encontrado em maior frequência no grupo de peso normal asmático comparado com o grupo de peso normal não asmático (*p*=0,02) e o alelo *Glu27* foi mais frequentemente encontrado no grupo de sobrepeso asmático comparado com o grupo de peso normal não asmático (*p*=0,03).

**Conclusões:**

A associação do alelo *Arg16* com a ocorrência de asma e a associação do alelo *Glu27* com adolescentes com sobrepeso asmáticos evidenciam a contribuição do gene *ADBR2* para o desenvolvimento da obesidade e da asma.

## Introdução

Excesso de peso e asma são doenças crônicas que atingem prevalência e morbidade alarmantes^1^ e o excesso de peso tem sido associado ao surgimento de problemas respiratórios, como a asma.^2^ Embora a complexa interação entre a exposição ambiental e a predisposição genética ao excesso de peso e o desenvolvimento de asma não esteja bem definida, estudos mostraram que os fatores genéticos podem influenciar a susceptibilidade de desenvolver obesidade^3^ e a associação entre polimorfismos genéticos e a asma tem sido relatada.^4^

Os receptores adrenérgicos beta 2 (*ADRB2*) são encontrados em várias regiões do corpo, incluindo as células de gordura, os vasos sanguíneos, o coração e as vias aéreas.^5^ Esses receptores desempenham um papel importante na gênese da obesidade e na regulação do balanço energético. Eles são responsáveis pela estimulação da atividade lipolítica no tecido adiposo^6^ e pelo controle do músculo liso brônquico,^7^ por meio da relaxação e da broncodilatação do músculo liso das vias aéreas.^8^

Os polimorfismos *Arg16Gly* (rs1042713) e *Gln27Glu* (rs1042714) do gene *ADRB2* parecem estar relacionados com o desenvolvimento de sobrepeso, hipertensão, síndrome metabólica^9^^,^ 10 e exacerbações da asma.^11^^,^ 12 Eles estão associados a mudanças na atividade do sistema nervoso simpático e podem alterar a lipólise^9^ e a regulação metabólica e cardiovascular^13^ e diminuir a função pulmonar e a resposta broncodilatadora à terapia com β2-agonistas.^14^

Em crianças e adolescentes, a associação dos polimorfismos *Arg16Gly* e *Gln27Glu* com obesidade ou asma foi investigada separadamente, com resultados controversos;^15^^,^ 16entretanto, nenhum estudo avaliou as frequências desses polimorfismos em crianças e adolescentes, considerando a obesidade e a asma conjuntamente. A associação entre o polimorfismo *Gln27Glu* e a aptidão física foi observada apenas em indivíduos adultos.^17^

O objetivo do presente estudo foi investigar a associação dos alelos dos polimorfismos *Arg16Gly* e *Gln27Glu* do gene *ADRB2* com a ocorrência de asma e sobrepeso em crianças e adolescentes e verificar se esses polimorfismos influenciam as variáveis antropométricas, clínicas, do perfil lipídico e da aptidão física.

## Método

A amostra foi composta de 206 crianças e adolescentes de ambos os sexos, entre 10 e 16 anos, provenientes da Região Sul do Brasil, divididos em quatro grupos: sobrepeso asmático (n=39), sobrepeso não asmático (n=115) peso normal asmático (n=12) e peso normal não asmático (controle) (n=40). Os participantes foram voluntários do Ambulatório de Endocrinologia Pediátrica e escolas públicas de Curitiba, Paraná (Brasil).

Os critérios de inclusão para os participantes com sobrepeso foram IMC acima do percentil 85 da Organização Mundial da Saúde (OMS)^18^ e para os participantes asmáticos, diagnóstico de asma por meio de avaliação médica e histórico clínico, como recomendado pela Sociedade Brasileira de Pneumologia e Tisiologia (SBPT).^19^ Neste estudo os participantes não foram classificados quanto à severidade da asma. Os critérios de exclusão foram: presença de diabetes e uso de drogas anorexígenas ou outras que pudessem interferir com o controle de peso, com os níveis de insulina, a pressão arterial, a glicose ou o metabolismo lipídico.

Os participantes e seus responsáveis assinaram o Termo de Consentimento Livre e Informado, de acordo com o protocolo aprovado pelo Comitê de Ética em Pesquisa da Universidade Federal do Paraná (CEP/HC2460.067/2011-03).

A estatura foi medida em centímetros (cm) com um estadiômetro fixado à parede (com precisão de 0,1 cm) e o peso foi medido em quilogramas (kg) com uma balança digital (capacidade máxima de 150 kg e resolução de 100 g). O Índice de Massa Corporal (IMC), expresso em kg/m^2^, foi calculado e classificado de acordo com os pontos de corte para idade e sexo, conforme proposto pela Organização Mundial da Saúde.^18^ A circunferência da cintura (CC) foi medida em centímetros com uma fita antropométrica flexível e não extensível (precisão de 0,1 cm). As pressões arteriais sistólica (PAS) e diastólica (PAD) foram medidas após 10 minutos de repouso, com o indivíduo sentado e com o braço direito apoiado à altura do coração.

As amostras de sangue foram colhidas no laboratório clínico na manhã seguinte após 12 horas de jejum, para hemograma completo, medição de glicose, insulina, colesterol total (CT), lipoproteína de alta densidade (HDL-C), lipoproteína de baixa densidade (LDL-C) e triglicérides (TG). As concentrações de CT, HDL-C, TG (mg/dL) e glicose foram analisadas pelo método colorimétrico enzimático automatizado (CHOD-PAP) (Laboratório Merck, Darmstadt, Alemanha; Laboratório Roche, Indianapolis, IN, EUA).

A concentração de insulina em jejum foi medida por imunoensaio quimioluminescente em µU/mL. O *software* HOMA Calculator v2.2 foi usado para calcular a resistência à insulina e o teste Quantitative Insulin Sensitivity Check Index (QUICKI), descrito por Katz et al. (2000),^20^ foi usado para avaliar a sensibilidade à insulina.

A análise da aptidão aeróbica foi feita em esteira e as respostas ventilatórias foram medidas com um sistema de espirometria calibrado respiração a respiração (Parvo Medics, True Max 2400, Utah, EUA), que forneceu informações sobre o consumo de oxigênio (VO_2_), produção de dióxido de carbono (VCO_2_), ventilação pulmonar (VP) e razão de troca respiratória (RTR=VCO_2_/VO_2_). Essas variáveis foram monitoradas a cada 15s. A frequência cardíaca foi monitorada com um monitor de frequência cardíaca (Polar - modelo A1, Finlândia). Para garantir que os participantes tivessem atingido o consumo máximo de oxigênio (VO_2max_), pelo menos dois dos seguintes critérios foram observados: a) exaustão ou incapacidade de manter a velocidade desejada; b) QR>1,0; c) frequência cardíaca (FC) máxima >190bpm.

As amostras de sangue (n=100) foram submetidas à extração de DNA de leucócitos pelo método *salting-out* 21 e diluídas até uma concentração de 20ng/µL. As variantes foram genotipadas com o TaqMan SNP Genotyping Kit (Applied Biosystems) no dispositivo Realplex 2 Mastercycler (Eppendorf). A mistura de reação continha: 5,0µL de TaqMan Universal PCR Master Mix, 0,5µL de TaqMan SNP Genotyping Kit especificado, 2,5µL de água ultrapurificada e 2µL de DNA (20ng/µL). Quatro amostras de cada genótipo foram sequenciadas para validação do método.

Para verificar a normalidade de distribuição dos dados foi usado o teste de Kolmogorov-Smirnov. Os testes *t* e de Mann-Whitney foram usados para comparação de médias para as variáveis com e sem distribuição normal, respectivamente. As frequências alélicas entre os grupos foram avaliadas pelos testes de χ2, com o *software* Clump.^22^ A significância foi fixada em um valor de *p*<0,05 e a análise foi feita com o *software* Statistica for Windows v.10 (StatSoft Inc., Tulsa, EUA).

## Resultados

As frequências dos alelos dos polimorfismos *Arg16Gly* e *Gln27Glu* do gene *ADRB2* em cada um dos grupos são mostradas na Tabela 1. O alelo *Arg16* foi encontrado com uma frequência mais elevada no grupo de peso normal asmático quando comparado com o grupo de peso normal não asmático (*p*=0,02). Para o polimorfismo *Gln27Glu*, observou-se uma frequência significativamente maior do alelo *Glu27* no grupo de sobrepeso asmático quando comparado com o grupo de peso normal não asmático (*p*=0,03) e uma tendência para frequência significativamente maior no grupo de sobrepeso asmático quando comparado com o grupo de sobrepeso não asmático (*p*=0,05).

**Tabela 1:** Frequências alélicas do polimorfismo *Arg16Gly* e *Gln27Glu* do gene *ADRB2* entre os grupos

Alelos	Sobrepeso/ asmáticos	Sobrepeso/não asmáticos	Peso normal/asmáticos	Peso normal/não asmáticos
	n (%)	n (%)	n (%)	n (%)
*Arg16Gly*				
*Arg 16*	26 (37%)	60 (39%)	12 (60%)^a^	22 (31%)
*Gly 16*	44 (63%)	92 (61%)	8 (40%)	48 (69%)
Total	70 (100%)	152 (100%)	20 (100%)	70 (100%)

*Gln27Glu*				
*Gln 27*	22 (50%)	98 (66%)	13 (65%)	41 (71%)
*Glu 27*	22 (50%)^b^	50 (34%)	7 (35%)	17 (29%)
Total	44 (100%)	148 (100%)	20 (100%)	58 (100%)

a Frequência significativamente maior em comparação com o grupo de peso normal não asmático (*p*=0,02).

b Frequência significativamente maior em comparação com o grupo de peso normal não asmático (*p*=0,03).

Os grupos avaliados (sobrepeso asmático, sobrepeso não asmático, peso normal asmático e peso normal não asmático) foram reunidos para verificar o possível efeito dos polimorfismos sobre as variáveis antropométricas, clínicas, do perfil lipídico e da aptidão física (exacerbações). Quando separados os grupos, de acordo com os genótipos do sítio *Arg16Gly*, em portadores da mutação (Arg Gly/Gly Gly) e portadores do genótipo usual (Arg Arg), as comparações das médias dos indicadores antropométricos, aptidão cardiorrespiratória, pressão arterial, variáveis de perfil bioquímico e metabólico não foram significativamente diferentes (Tabela 2).

**Tabela 2:** Médias ± DP das variáveis antropométricas, aptidão cardiorrespiratória, pressão arterial, bioquímicas e de perfil metabólico e comparações entre os portadores da mutação (Arg Gly/Gly Gly) e portadores de genótipo usual (Arg Arg), de acordo com os genótipos do sítio do polimorfismo *Arg16Gly*

Variáveis	Arg Arg	Arg Gly/Gly Gly	t ou Z	*p*‐valor
Idade	13,35±1,61	13,00±1,83	0,95	0,34
Peso (kg)	70,76±19,51	68,60±19,48	0,54	0,58
Estatura (cm)^a^	161,03±9,89	147,43±42,29	1,74	0,08
IMC (kg/m^2^)	26,91±5,89	26,86±6,65	0,03	0,97
IMC Z‐escore a	1,96±1,47	2,00±1,51	0,14	0,88
CC (cm)	91,11±17,19	89,94±16,93	0,33	0,73
FCRep (bpm)	79,88±9,18	80,15±12,17	−0,10	0,91
VO2máx (ml/kg/min)	36,46±7,81	35,16±7,91	0,68	0,49
PAS (mmHg)^a^	108,34±13,08	104,20±12,49	1,87	0,06
PAD (mmHg)^a^	70,68±11,51	68,34±10,27	1,09	0,27
CT (mg/dL)^a^	167,32±36,39	161,79±29,31	0,37	0,70
HDL‐C (mg/dL)^a^	47,55±9,73	49,95±12,43	−0,51	0,60
LDL‐C (mg/dL)^a^	95,34±31,42	88,69±26,20	0,70	0,48
TG (mg/dL)^a^	115,20±78,23	105,34±61,36	0,11	0,91
Glicose (mg/dL)	88,80±7,10	88,25±7,96	0,31	0,74
Insulina (mU/mL)^a^	12,29±9,20	12,92±9,97	−0,37	0,70
HOMA2‐IR^a^	1,57±1,16	1,63±1,21	−0,39	0,69
QUICKI^a^	0,35±0,04	0,35±0,04	0,39	0,69

IMC, índice de massa corporal; CC, circunferência da cintura; FCRep; frequência cardíaca em repouso; PAS, pressão arterial sistólica; PAD, pressão arterial diastólica; CT, colesterol total; HDL‐C, lipoproteína de alta densidade; LDL‐C, lipoproteína de baixa densidade; TG, triglicéride; HOMA2‐IR, Avaliação da homeostase metabólica; QUICKI, *Quantitative Insulin Sensitivity Check Index*; VO2max, consumo máximo de oxigênio.

a Variáveis sem distribuição normal.

Quando separados os grupos em portadores da mutação (Gln Glu/Glu Glu) e portadores de genótipo usual (Gln Gln), de acordo com os genótipos do sítio do polimorfismo *Gln27Glu*, a comparação da média e o DP das variáveis antropométricas, da pressão arterial, aptidão cardiorrespiratória e variáveis de perfil bioquímico e metabólico não apresentaram diferenças significativas, exceto por uma média significativamente mais elevada de CT observada no grupo portador do genótipo usual (Gln-Gln) (Tabela 3).

**Tabela 3:** Médias ± DP das variáveis antropométricas, aptidão cardiorrespiratória, pressão arterial, bioquímicas e de perfil metabólico e comparações entre os portadores de mutação (Gln Glu/Glu Glu) e portadores de genótipo usual (Gln Gln), de acordo com os genótipos do sítio do polimorfismo *Gln27Glu*

Variável	Gln Gln	Gln Glu/Glu Glu	t ou Z	*p*‐valor
Idade	13,16±2,02	13,29±1,64	−0,42	0,67
Peso (kg)	64,81±19,37	71,63±22,31	−1,91	0,05
Estatura (cm)^a^	151,74±33,15	148,43±45,50	1,33	0,18
IMC (kg/m^2^)	25,50±6,38	27,10±7,48	−1,34	0,18
IMC Z‐escore a	1,69±1,57	1,98±1,64	0,86	0,38
CC (cm)	86,96±17,44	92,38±19,12	−1,76	0,08
FCRep (bpm)	77,76±10,38	81,32±10,93	−1,73	0,08
VO2máx (ml/kg/min)	35,86±7,22	37,34±6,55	−1,03	0,30
PAS (mmHg)^a^	104,81±13,06	105,02±13,99	0,12	0,90
PAD (mmHg)^a^	70,62±10,59	66,98±10,76	−1,45	0,14
CT (mg/dL)^a^	169,23±34,93	158,48±31,51	−1,99	0,04^a^
HDL‐C (mg/dL)^b^	50,63±11,99	49,79±12,58	−0,10	0,91
LDL‐C (mg/dL)^b^	95,39±33,67	88,20±30,95	−1,40	0,16
TG (mg/dL)^b^	108,67±71,94	102,53±60,71	0,03	0,97
Glicose (mg/dL)	87,72±7,76	87,65±6,76	0,05	0,95
Insulina (mU/mL)^b^	11,85±10,72	12,46±7,94	1,08	0,27
HOMA2‐IR^b^	1,50±1,29	1,58±0,98	1,11	0,26
QUICKI^b^	0,36±0,05	0,34±0,04	−1,03	0,30

IMC, índice de massa corporal; CC, circunferência da cintura; FCRep; frequência cardíaca em repouso; PAS, pressão arterial sistólica; PAD, pressão arterial diastólica; CT, colesterol total; HDL‐C, lipoproteína de alta densidade; LDL‐C, lipoproteína de baixa densidade; TG, triglicéride; HOMA2‐IR, avaliação da homeostase metabólica; QUICKI, Quantitative Insulin Sensitivity Check Index; VO2max, consumo máximo de oxigênio.

a Diferença estatisticamente significativa.

b Variáveis sem distribuição normal.

## Discussão

A prevalência do sobrepeso na população brasileira entre 10 e 19 anos é de 20,5%^23^, enquanto que 10,3% das crianças e 13,8% dos adolescentes têm asma.^24^ Fatores genéticos foram demonstrados como contribuindo para o desenvolvimento da obesidade e asma durante a infância, mas os papéis de genes específicos e sua interação ainda são desconhecidos.^3^^,^ 4 ^,^ 15 Os polimorfismos *Arg16Gly* e *Gln27Glu* do gene *ADRB2* têm sido associados com o desenvolvimento de sobrepeso^9^ e as exacerbações da asma.^11^^,^ 12

As frequências da população anteriormente descritas para o alelo *Gly16* e para os alelos *Gln27* são 60,4% e 52,7%, respectivamente. 11 Em crianças e adolescentes obesos foram encontradas frequências semelhantes: 59% para o alelo *Gly16* e 62% para o alelo *Gln27*.^25^ Em um estudo recente, a frequência alélica para *Gly16* em crianças e adolescentes asmáticos foi de 55% e 83% para o *Gln27*.^26^

A maior frequência do alelo *Arg16* do polimorfismo *Arg16Gly*, observada no presente estudo no grupo asmático, quando comparado com o grupo de controle, sugere que a presença desse alelo pode estar associada com a ocorrência de asma. Observou-se uma tendência para uma frequência significativamente maior do alelo *Gly16* no grupo de sobrepeso asmático quando comparado com o grupo de peso normal asmático (*p*=0,06). Isso sugere que a presença do alelo *Gly16* pode estar relacionada com o sobrepeso em indivíduos asmáticos. Ellsworth et al.^15^ encontraram uma associação entre o polimorfismo *Arg16Gly* e a obesidade em participantes do sexo masculino: aqueles com genótipo Gly Gly mostraram um aumento do IMC durante a infância até a idade de jovens adultos. Resultados diferentes foram encontrados em meninas adolescentes. Isso mostra que as portadoras do genótipo Gly Gly têm uma probabilidade menor para obesidade do que aquelas com os genótipos Arg Gly ou Arg Arg (*p*=0,006) e que as meninas com genótipo Gly Gly têm menor IMC em comparação com aquelas com genótipo Arg Arg (*p*=0,049), mas superior em comparação com o genótipo Arg Gly (*p*=0,062).^16^ Em outro estudo, as participantes do sexo feminino portadoras do genótipo Arg Arg apresentaram maior IMC quando comparadas com aquelas com o genótipo Gly Gly.^27^ No presente estudo, não houve divisões entre os sexos devido ao pequeno tamanho da amostra; no entanto, nenhum outro estudo analisou grupos com sobrepeso e asma. Quando classificados pelo genótipo usual *versus* portadores da mutação do polimorfismo do sítio *Arg16Gly*, não foram encontradas diferenças para as variáveis antropométricas.

Para o sítio do polimorfismo *Gln27Glu*, foi observada uma frequência maior do alelo *Glu27* no grupo de sobrepeso asmático quando comparado com o grupo controle (*p*=0,03) e uma tendência à significância em comparação com o grupo de sobrepeso não asmático (*p*=0,05). Isso sugere que o alelo *Glu27*pode estar envolvido no desenvolvimento de asma em indivíduos com sobrepeso. Large et al.^9^ encontraram o alelo *Glu27* associado à obesidade e Ochoa et al.^28^ não encontraram associação entre o polimorfismo *Gln27Glu* e obesidade em meninos; mas encontraram um risco maior de obesidade em mulheres portadoras do alelo *Glu27*. A pesquisa mostrou que meninas negras portadoras do genótipo Glu/Glu têm maior média de CC do que as meninas sem o alelo, uma associação que não foi encontrada nos meninos. 29 No presente estudo, quando separados pelo genótipo usual e os portadores de mutação para o sítio do *Gln27Glu*, não foram encontradas diferenças para as variáveis antropométricas.

Há evidências de que variantes genéticas dos adrenorreceptores têm um papel na fisiopatologia da hipertensão, mas os resultados sobre a associação entre os polimorfismos *ADRB2* ainda são discordantes.^10^ Um estudo de Chou et al.^16^ encontrou associação do polimorfismo *Arg16Gly* com hipertensão em adolescentes obesos. Isso mostra que os portadores do genótipo Gly/Gly têm menor probabilidade de hipertensão do que os pacientes com genótipo Arg/Gly ou Arg/Arg (*p*=0,005). Nenhum outro estudo, até o momento, associou polimorfismos do gene *ADRB2* e pressão arterial em crianças e adolescentes. No presente estudo não foram encontradas evidências de que os polimorfismos avaliados estejam agindo sobre as variáveis da pressão arterial.

Os receptores adrenérgicos desempenham um papel importante na regulação da lipólise e do gasto energético, por isso é possível que os polimorfismos nesses genes contribuam para o aparecimento de alterações metabólicas.^30^ As pesquisas brasileiras com crianças obesas não encontraram diferenças nas variáveis metabólicas e bioquímicas (glicose, CT, LDL-C, HDL-C, TG, leptina, insulina, área de glicose e insulina e HOMA-IR) entre haplótipos para os polimorfismos *Arg16Gly* e *Gln27Glu*.^13^ No presente trabalho, quando separados os grupos por genótipo usual *versus* portadores de mutação no sítio do polimorfismo *Arg16Gly*, não foram encontradas diferenças para as variáveis metabólicas e bioquímicas. Para o sítio do polimorfismo *Gln27Glu*, o grupo usual apresentou maior valor médio de CT quando comparado com o grupo portador de mutação.

A associação do alelo *Arg16* com a ocorrência de asma e o alelo *Glu27* com o desenvolvimento de asma em indivíduos com sobrepeso demonstrou a sua contribuição para o desenvolvimento da obesidade e da asma durante a infância e a adolescência. A possível associação entre os polimorfismos estudados e o gênero, bem como a gravidade da doença, não pôde ser avaliada, uma vez que a divisão dos pacientes em quatro grupos reduziu o número de cada um, o que constituiu a principal limitação do estudo. Novos estudos com amostras maiores podem permitir a separação dos grupos por gênero e a implantação de análise estatística mais consistente em relação aos papéis dos alelos *Arg16* e *Glu27* no sobrepeso/obesidade e na asma na infância. Os polimorfismos avaliados parecem não influenciar a aptidão física na infância e na adolescência.
